# Sex Steroid Priming on Growth Hormone Stimulation Test: A Scoping Review

**DOI:** 10.3390/children12101286

**Published:** 2025-09-24

**Authors:** Ignazio Cammisa, Donato Rigante, Giorgio Sodero, Clelia Cipolla

**Affiliations:** 1 Department of Life Sciences and Public Health, Fondazione Policlinico Universitario A. Gemelli IRCCS, 00168 Rome, Italyclelia.cipolla@policlinicogemelli.it (C.C.); 2 Department of Pediatrics, Università Cattolica del Sacro Cuore, 00168 Rome, Italy; 3 Pediatric Department, Perrino Hospital, 72100 Brindisi, Italy; 4 Pediatric Endocrinology Unit, Perrino Hospital, 72100 Brindisi, Italy

**Keywords:** growth hormone deficiency, growth hormone stimulation test, sex steroid priming, testosterone, estrogen, diagnostic accuracy, personalized medicine

## Abstract

**Highlights:**

**What are the main findings?**

- Sex steroid priming before GH stimulation testing increases GH peak responses in many peripubertal children, reducing the risk of false-positive GHD diagnoses.
- Both estrogen (girls) and testosterone (boys) enhance spontaneous and stimulated GH secretion, though effects vary with dose, duration, route, and individual factors.

**What is the implication of the main finding?**

- Priming may improve the diagnostic accuracy of GH testing and help distinguish true GHD from temporary low GH responses due to low sex steroid levels.
- Standardized priming protocols and inclusion of additional biomarkers (IGF-1, IGFBP-3, adipokines, and kisspeptin) could optimize clinical decision-making. Future studies should focus on multicenter trials, patient-centered outcomes, and predictive algorithms to guide individualized management.

**Abstract:**

**Background/Objectives:** Growth hormone (GH) secretion and activity are closely modulated by sex steroids, particularly during puberty, when GH response to stimulation tests (GHSTs) may be blunted in children with low circulating sex steroids. This systematic review evaluates the role of sex steroid priming in enhancing GH secretion during GHST and improving diagnostic accuracy especially in peripubertal children with GH deficiency (GHD). **Methods**: We conducted a medical literature review to assess the use of sex steroid priming in children undergoing GHST; our search was carried out via PubMed after paper selection based on strict eligibility criteria. **Results**: Seventeen studies published between 1969 and 2022 were analyzed overall, which corresponded to a total number of 1097 participants. Most studies reported a significant increase in GH peak levels after priming, reducing false-positive GHD diagnoses. However, protocols varied considerably in hormone type, dosage, and administration, and some studies showed no benefit in the case of a true GHD. **Conclusions**: While sex steroid priming appears to improve GHST specificity in GHD without negatively impacting growth outcomes, standardized guidelines about pre-administration of exogenous sex steroids are currently lacking, and further prospective studies are needed to define its diagnostic potential.

## 1. Introduction

Growth hormone (GH), along with its primary mediator insulin-like growth factor 1 (IGF-1), plays a seminal role in regulating a child’s linear growth: it acts through both systemic endocrine mechanisms and localized autocrine and paracrine signalling pathways [1]. GH is secreted in a pulsatile manner by somatotroph cells in the anterior pituitary, and its release is tightly controlled by opposing hypothalamic signals: growth hormone-releasing hormone (GHRH) stimulates secretion, while somatostatin inhibits it [1]. In addition, peripheral factors such as sex steroids further enhance GH secretion. The interplay between the GH axis and sex steroids is profoundly complex. Sex steroids enhance the sensitivity of somatotroph cells in the anterior pituitary to GHRH, thereby potentiating GH secretion. Conversely, GH facilitates pubertal development by stimulating the local synthesis of insulin-like growth factors (IGFs) within the gonads and by augmenting gonadal responsiveness to gonadotropins [2,3]. Sex steroids modulate GH secretion and function through both direct and indirect signalling effects via endocrine and paracrine pathways [4]. Estrogens act at the hypothalamus and pituitary levels, where they promote GH release by suppressing somatostatin activity, increasing the expression of GHRH receptors, and enhancing GH secretion in response to ghrelin. Concurrently, testosterone supports this process by boosting GH-induced IGF-1 synthesis and by increasing the expression of GH receptors, thereby enhancing GH signalling [4,5,6]. The complex interplay between the two axes is evidenced by data indicating that GH secretion remains relatively low in early puberty, with a significant increase observed during mid-to-late pubertal stages. This increase is mainly driven by a rise in GH pulse amplitude, which closely parallels the progressive elevation of circulating sex steroid levels throughout puberty [7,8,9]. Notably, spontaneous 24 h GH secretion progresses through the pubertal stages, peaking earlier in girls—sometimes preceding overt clinical signs of puberty—and later in boys, corresponding with their period of maximal growth velocity [9]. Based on this evidence, it is important to acknowledge that peripubertal children with low circulating sex steroid levels may show an attenuated response to GH stimulation tests (GHSTs) compared to age-matched peers who have entered puberty. In such instances, priming—the pre-administration of exogenous sex steroids—may be used to increase pituitary sensitivity and optimize GH secretion during testing [10]. Several studies have proposed the use of sex steroid priming in short prepubertal children—particularly those with a predicted adult height below -2 SDS and more specifically in boys over 11 years and girls over 10 years—as a tool to better differentiate constitutional delay of growth and puberty (CDGP) from other conditions and to reduce the risk of unnecessary recombinant GH therapy. However, a clear consensus regarding the routine implementation of priming prior to assessment of the GH–IGF-1 axis has yet to be established [10,11].

The aim of this review was to evaluate the role of sex steroid priming in GHST by analyzing current evidence in the medical literature and assessing its impact on diagnostic accuracy for GH deficiency.

## 2. Materials and Methods

We performed a literature review to evaluate the use of priming with sex steroids in children undergoing GHST.

### 2.1. Review Questions

The main research question of our scoping review was “What is the impact of sex steroid priming in children undergoing GHST?”. Additionally, we analyzed the following secondary questions:

- Does the route of administration of sex steroids influence the outcomes of GHST?
- Is there consistency in the age at which sex steroid priming is applied and in the methods used for priming?

### 2.2. Inclusion Criteria

Studies were considered eligible for inclusion if they met the following criteria: (1) involved children undergoing GH stimulation testing; and (2) included the administration of sex steroid priming. The following studies were excluded: (1) non-English language publications; (2) studies that did not evaluate priming protocols.

### 2.3. Search Strategy

**Figure 1:**
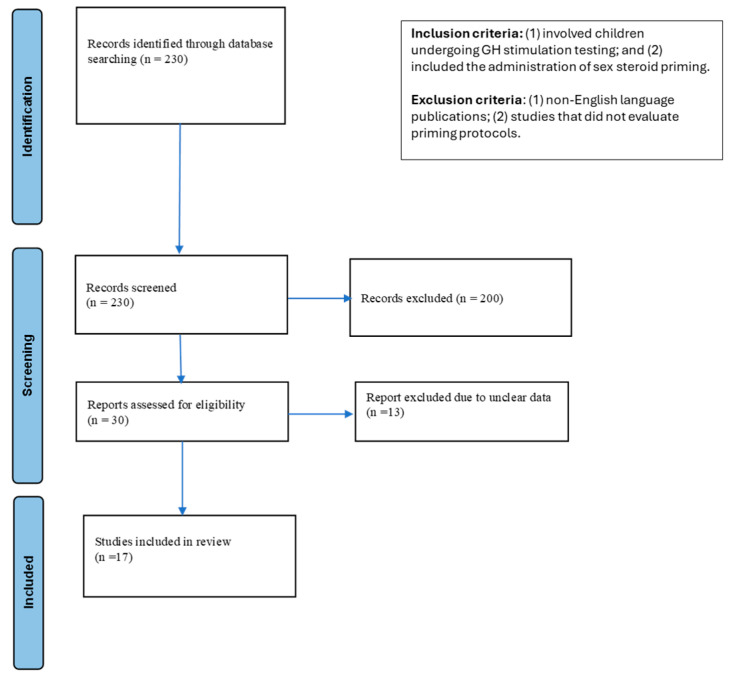
PRISMA 2020 flow diagram for new systematic reviews which included searches of databases and registers only.

We performed the scoping review without data restrictions, in the bibliographic database PubMed using the following keywords: “priming & growth hormone”, “priming & GH”, “priming & GH stimulation test”, “priming & children”, “sex steroid & growth hormone”, “testosterone & growth hormone”, “estrogen & growth hormone”. This scoping review was conducted following the Preferred Reporting Items for Systematic Reviews and Meta-Analyses (PRISMA) guidelines to ensure transparency and completeness in reporting. Abstracts were independently screened by two reviewers (IC and CC), who rigorously applied the predefined inclusion and exclusion criteria to determine the eligibility for full-text review. All studies meeting the eligibility criteria were subsequently reviewed in full, and any disagreements between the reviewers were resolved through open discussion. Our screening encompassed randomized as well as non-randomized controlled trials. We also considered all forms of observational research, both prospective and retrospective, including case–control, cohort, and cross-sectional studies, as well as smaller case series and individual case reports. Our search strategy is described in Figure 1.

### 2.4. Study Selection

A total of 230 records were initially identified through the PubMed database. In the first screening phase, we excluded 50 articles that were not in English, 30 records with unavailable full texts, and 100 duplicate entries. In the second phase, 20 records were excluded after reviewing titles and abstracts, as they did not meet the previously defined inclusion criteria. Of the remaining 30 studies, 13 were excluded after further discussion regarding the reliability of their data. As a result, 17 articles were selected and included in this review. The detailed selection process is illustrated in a PRISMA flow diagram, while a comprehensive summary of the findings is presented in Table 1.

### 2.5. Data Extraction

Data extraction was conducted under the close supervision of the lead investigator (CC). For each eligible study, the following information was collected: study design, characteristics of the study population, details of the priming protocol, and the type of GH stimulation test employed. Additionally, we assessed reported study limitations and disclosed conflicts of interest if available. A formal ethical approval was not required for this review.

### 2.6. Data Analysis and Presentation

We adhered to the Preferred Reporting Items for Systematic Reviews and Meta-Analyses extension for Scoping Reviews (PRISMA-ScR) Checklist (see Supplementary Materials). A narrative synthesis was undertaken to summarize and interpret the findings of the included studies.

## 3. Results

This review included a total of 17 studies published between 1969 and 2022 [11,12,13,14,15,16,17,18,19,20,21,22,23,24,25,26,27]. The studies varied in design and included: 4 randomized controlled trials, 7 controlled studies, 5 retrospective studies, and 1 clinical trial. Sample sizes ranged widely, from as few as 3 to as many as 246 participants, with an overall combined sample of 1097 children. Priming protocols were different in dosages and time. Estrogen priming was used in 11 studies, with doses ranging from 20–50 micrograms/m^2^/day for 2–5 days to 1–2 mg/day of oral estrogen for 2–3 days. Some protocols included 2.5 mg administered the evening before and the morning of GHST. Testosterone priming was employed in further 10 studies, with regimens including: 25 mg to 200 mg, administered as intramuscular injections (single doses of 100 mg, repeated monthly for 3–4 months, or 50 mg every 4 weeks for 3 months), as oral (120 mg twice a day for 5 days) and transdermal testosterone (2% gel, 10 mg/day for 3 months). Several studies included both estrogen and testosterone, depending on the sex of the participants. A variety of GH stimulation agents were utilized: arginine was the most frequently used, included in 10 of the 17 studies, while clonidine was used in 8 studies, followed by ITT in 7 studies, levodopa in 3 studies, glucagon in 3 studies, and GHRH in 1 study. Many studies combined two or more agents for stimulation. A clear improvement in the GH peak response after priming was reported in 13 studies. Conversely, a lack of significant improvement was reported by 4 studies.

## 4. Discussion

Children entering the peripubertal stage who exhibit short stature or fail to experience the expected pubertal growth spurt require a thorough endocrine assessment [10]. In these patients, it is essential to distinguish GHD from other conditions such as CDGP, idiopathic short stature (ISS), and various other causes of short stature [10,28]. Diagnosing GHD in children is a complex process that involves a thorough clinical evaluation alongside auxological evaluation and biochemical testing of the GH-IGF axis or imaging studies [29,30,31,32]. According to the current guidelines, the most important component of the diagnostic flowchart, though not sufficient on its own, is the use of GHST with pharmacological agents. However, these stimulation tests are not standardized according to age, pubertal stage, body mass index (BMI), or the particular drug used, even though there is strong evidence showing that these variables significantly influence the GH response [29,30,31,32]. Making an accurate diagnosis of GHD is crucial, as appropriate treatment with recombinant human growth hormone (r-GH) can help affected children reach their full adult height potential [33,34,35]. In light of these findings, enhancing the diagnostic accuracy of GHST is essential, particularly in children at risk of false-positive results, such as those in the peripubertal phase, where GH secretion may be closely linked to sex steroid hormone levels [2,3,4,5]. Several studies from past decades have shown that about half of these children—initially diagnosed with low GH secretion and treated with GH—demonstrated normal GH responses upon retesting after treatment cessation [36,37,38,39]. Furthermore, untreated children often experienced accelerated growth during puberty, accompanied by increases in both spontaneous and stimulated GH secretion, suggesting that the reduced GH levels seen during the peripubertal period are more likely due to a deficiency of sex steroids rather than to a true GH deficiency [36,37,38,39]. Thus, the transiently reduced response to GHST in peripubertal children with low peak GH levels may lead to unnecessary GH treatment, as these levels often normalize with the progression of puberty [31]. Not all children diagnosed with GHD during childhood produce sufficient amounts of GH in adulthood, although some may potentially do so. Therefore, these results likely reflect normal growth and physiological maturation rather than inaccuracies in the initial testing.

Sex hormone priming before GHST was introduced in 1968 to boost GH secretion and help distinguish between true GHD and CDGP [3,40]. However, there is still no agreement on which patients should receive this procedure, the optimal timing, the most appropriate dosages or type of sex steroids to use, and methods of administration [3]. Moreover, since the physiological increase in GH secretion during puberty generally occurs in boys with a bone age of 12–14 years and testicular volume of 10–15 mL, and in girls with a bone age of 11–13 years and breast development at Tanner stage 3, peripubertal children still exhibit relatively low levels of endogenous sex steroids [41,42,43,44]. As a result, several researchers have suggested that the seemingly normal GH response observed after sex steroid priming during stimulation tests may be temporary and does not represent the sustained GH production necessary for normal pubertal growth. Consequently, this may lead to underdiagnosis of transient GHD in peripubertal children who might otherwise benefit from r-GH therapy [41,42,43,44].

The use of sex steroid priming remains characterized by manifold modalities with considerable variability in its application across countries and clinical settings [3]. A survey by Wyatt et al., involving 251 pediatric endocrinologists in the United States of America, revealed that only one-third used priming in peripubertal children [45]. Similarly, data from the European Society for Paediatric Endocrinology (ESPE) indicated that priming was employed by 50.2% of respondents for boys and 40.9% for girls [46]. These findings highlight the ongoing lack of consensus regarding the routine use of sex steroid priming in clinical practice.

Our comprehensive review of 17 studies, spanning over five decades (1969–2022), provides an updated synthesis of the available evidence on the utility of priming with estrogen in girls and testosterone in boys prior to GHST, particularly in peripubertal children with suspected GHD. The specific age at which priming should be administered remains one of the most heterogeneous aspects. The 2001 ESPE survey reported a mean age of priming of 10.6 ± 0.18 years for boys and 9.24 ± 0.21 years for girls. While some endocrinologists begin priming at around 9 years of age for boys and 8 years for girls, others reserve it for patients with clear signs of delayed puberty, typically around 13–14 years in boys and 11–12 years in girls [7]. An Audit of National Guidelines and Practice involving many countries (Spain, France, Poland, United Kingdom, Netherlands, Denmark, Italy, and United States of America) indicated that sex steroid priming is typically applied in boys aged 10 to 13 years and girls aged 8 to 12 years, while the Pediatric Endocrine Society Guidelines recommended priming for prepubertal boys older than 11 years and prepubertal girls older than 10 years who have a predicted final height greater than -2 SD compared to the reference population [47,48]. Consistent with these recommendations, our review found that the age of children undergoing sex steroid priming ranged from 7.4 to 15.4 years.

The type, dosage, and timing of sex steroids used for priming also differ between centers, with substantial variability in protocols. The most frequently employed methods include, for boys, an intramuscular injection of 100 mg testosterone depot given 7 to 10 days prior to the planned GHST while for girls estradiol is typically administered orally for 2–3 days before the GHST, either as a single daily dose of micronized estradiol valerate (1 mg for children weighing up to 20 kg and 2 mg for those over 20 kg) or as ethinylestradiol given twice daily at a dose of 40 µg/m^2^/day [7]. Actually, various estrogen and testosterone preparations are available, each with distinct pharmacokinetic profiles depending on formulation strength and route of administration [49,50]. For instance, oral ethinylestradiol produces a rapid rise in IGF-1 levels and reaches peak plasma concentrations within 0.5 to 1.5 h, with an elimination half-life of approximately 12–14 h. In contrast, transdermal estrogen formulations typically result in lower systemic levels of estrogen metabolites [49,50]. However, Borghi et al. demonstrated that estrogen patches provide a safe and feasible alternative, delivering a continuous release of estradiol and maintaining stable plasma concentrations over a 72 h period [22]. Simultaneously, Mastromattei et al. proposed the use of transdermal testosterone gel formulations, whereas Drop et al. employed oral testosterone administration in their study, despite intramuscular injection remaining the most used route [13,27].

In our review, the dosages of sex hormones appeared highly variable and showed little consistency across studies, complicating direct comparisons between studies. Among the 11 studies utilizing estrogen priming, doses ranged from low microgram-per-square-meter regimens to fixed milligram doses administered over different time frames. For instance, Bacon et al. employed a relatively high dose of 5 mg of oral estrogen twice daily for 3 days, while Drop et al. used 50 mcg twice daily for 5 days [12,13]. Ross et al. opted for 1 mg of oral estrogen twice daily for 2 days, whereas Wilson et al. administered a 2.5 mg bolus dose the evening before and the morning of the GH stimulation test, demonstrating the diversity even among short-course protocols [15,17]. Other studies utilized weight-based dosing: for example, Moll et al. used 20–40 mcg/m^2^ of ethinylestradiol, while Marin et al. used 40 mcg/m^2^/day for 2 days [14,18]. Similarly, testosterone priming protocols were highly variable. Several studies used intramuscular injections, such as Chalew et al., who administered 200 mg once monthly for 4–5 months, and Muller et al., who used a single 100 mg testosterone IM injection 3–10 days before testing [16,20]. Couto-Silva et al. compared boys receiving two versus four doses of 100 mg testosterone, showing that the four-dose group had significantly higher GH peaks [21]. Nevertheless, our review indicates that while high-dose and prolonged priming regimens are generally associated with more substantial increases in GH peak levels, lower-dose protocols also resulted in appreciable GH responses in a significant number of cases. For example, Couto-Silva et al. showed an increased mean peak GH response to arginine-insulin stimulation after receiving four doses of 100 mg intramuscular testosterone (21.3 ± 2.0 μg/L), compared to those receiving two doses (14.7 ± 1.7 μg/L; *p* = 0.04). On the other hand, Moll et al. noted similar responses between one and two days of estrogen treatment, though specific data were not provided [14,21].

A key finding across many studies included in this review is the significant increase in GH peak levels following sex steroid priming. Several studies reported that initially subnormal GH responses rose above diagnostic thresholds after the administration of testosterone or estrogen. However, the extent of this effect appears to be modulated by dosage, duration, and route of hormone administration. One of the earliest studies by Bacon et al. demonstrated that administering estrogen (5 mg twice daily for three days) resulted in GH levels comparable to those induced by arginine stimulation alone [12]. Similarly, Moll et al. found that all children receiving at least 20 μg/m^2^ of ethinylestradiol exhibited sufficient GH responses to levodopa, indicating a likely dose–response relationship [14]. Ross et al. also reported a significant rise in GH concentrations following estrogen priming (1 mg twice daily for two days), with mean values increasing from 3.2 to 7.7 mU/L (*p* = 0.002) [15]. Marin et al. in a larger randomized trial observed that estrogen administered at 40 μg/m^2^ for two days broadened the range of normal GH peaks, elevating the upper limit to 40.5 μg/L [18]. Further supporting this effect, Martinez et al. found that estrogen administration increased GH peaks from 17.8 ± 10.9 µg/L to 27.9 ± 14.5 µg/L in children with idiopathic short stature, while Borghi et al. noted a significant rise in median GH levels from 16.6 to 22.0 ng/mL following a three-day course of 50 μg/day ethinylestradiol [19,22]. Contemporary, Chalew et al. observed a near doubling of the mean 24 h integrated GH concentration—from 1.7 to 3.3 μg/L—after four to five monthly intramuscular injections of 200 mg testosterone [16]. Muller et al. reported that 77% of participants exhibited increased GH peaks after a single 100 mg injection, in contrast to uniformly subnormal baseline values (<10 ng/mL) [20]. In a retrospective cohort, Couto-Silva et al. found that four 100 mg testosterone doses significantly enhanced GH peaks (21.3 ± 2.0 µg/L), compared to only two doses (14.7 ± 1.7 µg/L; *p* = 0.04) [21]. Similar findings were described by Molina et al., who found post-priming GH peaks increased to 12.32 ± 8.7 µg/L from a pre-priming baseline of 4.87 ± 2.72 µg/L in over half of the participants [24]. Recently, Lennartsson et al. conducted a retrospective chart review including 132 children with short stature who underwent both nocturnal spontaneous GH secretion assessment and an arginine–insulin stimulation test. Compared with non-primed children, those who were primed exhibited significantly higher peak and mean spontaneous GH concentrations (14.2 μg/L vs. 10.8 μg/L, and 3.0 μg/L vs. 2.3 μg/L, respectively). Divergent results between spontaneous and stimulated GH tests were less frequent among primed children (4%) than in non-primed children (23%) [51]. Despite these generally favorable findings, some studies have yielded inconsistent outcomes. For instance, Drop et al. and Wilson et al. observed no significant change in GH peaks following sex hormone priming, and Soliman et al. reported only a marginal increase in the proportion of patients classified as normal responders (52% with priming versus 47% without) [11,13,17]. Taken together, these mixed results suggest that while sex steroid priming can enhance GH secretion in many cases, its clinical impact may depend on the individual patient’s characteristics, priming regimens, and test protocols.

Despite this supportive evidence, a consensus on priming protocols is currently lacking, and the differences in hormonal types, dosages, timing, and GHST hinder any development of universal guidelines.

## 5. Limitations

While this review provides an overview of current evidence regarding the impact of sex steroid priming on GHST, several limitations should be acknowledged. The medical literature search may have missed relevant studies not indexed on PubMed or not captured by the selected keywords that were used. Moreover, considerable heterogeneity was observed across studies in terms of population characteristics (age, growth parameters, and pubertal staging), definitions of pubertal status, GHST protocols, and priming regimens. Differences in the type, dose, and duration of sex steroid administration, as well as in the agents used for stimulation, complicate direct comparisons. Additionally, inconsistencies in GH peak reporting and the use of variable thresholds to define a normal response (7 µg/L versus 10 µg/L) further limit comparability. As a result of this methodological variability, subgroup analyses by specific test protocols, priming strategies, or age groups were not feasible. Future well-designed large-scale studies are needed to clarify these effects. While we conclude that additional data are needed and randomized controlled trials with adequate statistical power would be ideal, conducting such studies in this context is challenging. Each patient would effectively need to serve as their own control, requiring two growth hormone stimulation tests—one with and one without sex hormone priming. Given the ethical considerations in pediatric research, the potential risks associated with this approach likely outweigh the anticipated benefits.

## 6. Future Directions and Conclusions

In conclusion, the available evidence suggests that sex steroid priming may enhance GH peak responses in GHST and thereby reduce false-positive results, particularly in peripubertal children. However, the findings across studies are inconsistent, and the clinical utility of priming remains uncertain. Current data are insufficient to support routine implementation in practice, especially given the lack of robust evidence in specific subgroups such as children with Noonan syndrome, SGA, obesity, or chronic conditions. Further well-designed, adequately powered, multicenter randomized controlled trials are needed to clarify the role of priming. Such studies should focus on standardizing protocols (including type, dose, duration, and timing of steroid administration), establishing diagnostic cut-offs tailored to age, sex, and pubertal stage, and evaluating both cost-effectiveness and long-term outcomes (e.g., final height, psychological health, and quality of life). Incorporating a broader range of biomarkers (e.g., IGF-1, IGFBP-3, adipokines, kisspeptin, and proteomic or metabolomic profiles), alongside clinical, growth, and imaging data, may further refine diagnostic accuracy and patient stratification. Additionally, patient-centered outcomes such as acceptability, adherence, and safety require systematic assessment. While priming holds potential, clinicians should remain cautious until stronger evidence becomes available, and the development of standardized protocols and predictive tools will be essential before clinical practice can be meaningfully informed.

## Figures and Tables

**Table 1:** List of studies dealing with sex steroid priming in growth hormone stimulation tests.

Study	Study Design	Sample Size (N)	Mean Age (Years)	Priming Protocol	GHST	GH Peak Cut-Off	Outcomes
Bacon et al. (1969) [12]	Control study	26	NA	Estrogen (5 mg twice a day for 3 days)	Arginine	NA	Estrogen stimulated GH secretion to a similar extent as the arginine test.
Drop et al. (1982) [13]	Control study	8	NA	Estrogen (50 mcg twice a day for 5 days) Testosterone (120 mg twice a day for 5 days)	Arginine, ITT	8 mU/L	The peak GH response after priming remained abnormally low and showed no significant change compared to earlier test results.
Moll et al. (1986) [14]	Control study	23	12.3 ± 1.4	Estrogen (20 to 40 µg/m^2^)	Levodopa	7 ng/mL	18/23 children exhibited a positive response to levodopa prior to estrogen administration. 20/23 children who received priming demonstrated an adequate GH response.
Ross et al. (1987) [15]	Control study	14	NA	Estrogen (1 mg twice a day for 2 days)	ITT, GHRH	NA	Baseline GH levels were significantly increased following priming; the average GH level was 3.2 mU/L in unprimed tests, compared to 7.7 mU/L after priming (*p* = 0.002).
Chalew et al. (1988) [16]	Control study	8	NA	Testosterone (200 mg once a month for 4–5 months)	Arginine, Clonidine, ITT, Levodopa	10 µg/L	Before priming, the average 24 h IC-GH concentration was 1.7 ± 1.0 micrograms/L. After testosterone, the IC-GH increased to 3.3 ± 2.6 micrograms/L.
Wilson et al. (1993) [17]	RCT	65	8.8	Estrogen (2.5 mg the evening before and the morning of the GHST)	Clonidine	10 μg/L	No statistically significant differences were observed in mean GH levels between children who underwent sex steroid priming and those who did not.
Marin et al. (1994) [18]	RCT	84	NA	Estrogen (40 µg/m^2^ daily for 2 days)	Arginine, ITT	7 μg/L	Estrogen administration increased the normal range of peak GH responses from 1.9–20.3 to 7.2–40.5 micrograms/L.
Martinez et al. (2000) [19]	RCT	59	10.7 ± 3.3	Estrogen (1 or 2 mg for 3 days)	Arginine, Clonidine	NA	In children with ISS, the peak GH response was 17.8 ± 10.9 µg/L under placebo and increased to 27.9 ± 14.5 µg/L following estrogen administration. No significant enhancement in GH secretion was observed in children with GHD after estrogen.
Muller et al. (2004) [20]	Clinical trial	26	14.3 ± 1.1	Testosterone (100 mg for 3–10 days)	Arginine	10 ng/mL	In all 26 patients, the peak GH level following the initial arginine stimulation test was under 10 ng/mL (average 5.6 ± 2.6 ng/mL). After priming 20 patients (77%) showed an increased GH peak.
Couto-Silva et al. (2005) [21]	Retrospective study	148	15.0 ± 0.1	Testosterone	Arginine, ITT	10 μg/L	A GH peak below 10 µg/L was observed in 8 out of 32 cases when the GHSTs were performed with testosterone heptylate priming and in 62 out of 153 cases without priming. Among the boys who received two doses of 100 mg testosterone, 7 out of 11 had low GH peaks (14.7 ± 1.7 µg/L), compared to only 1 out of 21 in the group that received four doses of 100 mg (21.3 ± 2.0 µg/L, *p* = 0.04).
Borghi et al. (2006) [22]	Control study	22	8.8	Estrogen (50 µg/day)	Clonidine	10 pg/mL	The median GH peak rose significantly following priming (from 16.6 ng/mL to 22.0 ng/mL).
Gonc et al. (2008) [23]	Retrospective study	50	13.2 ± 1.7	Testosterone (62.5 mg/m^2^ or 125 mg/m^2^)	Levodopa	10 ng/mL	In the low-dose group, the average peak GH level rose from 4.9 ± 3.0 to 19.3 ± 5.9 ng/mL. In the conventional-dose group, it increased from 5.4 ± 2.1 to 17.0 ± 5.9 ng/mL. No statistically significant differences in the mean peak GH levels among the three groups, either before or after priming (*p* = 0.819).
Molina et al. (2008) [24]	Control study	39	12.37 ± 2.24	Estrogen (1 mg daily for 3 days) Testosterone (100 mg for 5–8 days)	Clonidine	10 μg/L	After receiving priming, 21 out of 39 children (53.8%) showed an increase in GH levels to above 10 µg/L. The average peak GH level following priming rose to 12.32 ± 8.7 µg/L, compared to 4.87 ± 2.72 µg/L before priming.
Soliman et al. (2014) [11]	RCT	92	12	Estrogen (1.25 mg for 3 days) Testosterone (25 mg for 7–10 days)	Clonidine	7 ng/mL	Priming with sex steroids did not lead to a significant increase in the proportion of patients showing a normal GH response (52% with priming compared to 47% without).
Sato et al. (2020) [25]	Retrospective study	3	13.9–14.6	Testosterone (100 mg)	Arginine, Glucagon, ITT	6 ng/mL	GH peak levels were higher when testosterone priming was used.
Galazzi et al. (2021) [26]	Retrospective study	184	12.4 ± 2.08	Estrogen Testosterone	Arginine, Clonidine, Glucagon, ITT	8 μg/L	Priming before provocative testing for GH reserve appears to enhance the accuracy of diagnosing GH deficiency.
Mastromattei et al. (2022) [27]	Retrospective study	246	14.1	Testosterone (50 mg every 4 weeks for 3 months or transdermal 2% 10 mg daily for 3 months)	Arginine, Clonidine, Glucagon	8 µg/L	A positive result indicating possible GHD was found in 31 of 107 subjects (29%), with most positive tests (28 out of 31, or 90%) occurring in those who had not received sex hormone priming.

Growth hormone (GH), Growth Hormone Releasing Hormone (GHRH), Growth hormone deficiency (GHD), Growth hormone stimulation test (GHST), Idiopathic short stature (ISS), Integrated growth hormone (IC-GH), Insulin tolerance test (ITT), Idiopathic short stature (ISS), Randomized controlled trial (RCT).

## Data Availability

Not applicable.

## Supplementary Materials

The following supporting information can be downloaded at: https://www.mdpi.com/article/10.3390/children12101286/s1, Preferred Reporting Items for Systematic reviews and Meta-Analyses extension for Scoping Reviews (PRISMA-ScR) Checklist, from [52].
